# A Rare Case Reveals Important Consideration of the Diagnosis of Giant Cell Arteritis in Patients with Bilateral Painful Optic Perineuritis

**DOI:** 10.3390/reports9020187

**Published:** 2026-06-15

**Authors:** Jordan Santos, Faraz Behzadi, S. Mozammil Alam, Thomas C. Varkey, David C. Maeng, Ghassan J. Ibrahim, Trent H. Smith, Alan Wang

**Affiliations:** 1 Department of Neurology, University of Arizona College of Medicine, Phoenix, AZ 85004, USA; jordan.santos3@bannerhealth.com (J.S.);; 2 Department of Pathology, University of Arizona College of Medicine, Phoenix, AZ 85004, USA; 3 Department of Rheumatology, University of Arizona College of Medicine, Phoenix, AZ 85004, USA

**Keywords:** optic perineuritis, giant cell arteritis, acute vision loss, painful extraocular eye movements, case report

## Abstract

**Background and Clinical Significance:** Giant cell arteritis (GCA) is an autoimmune vasculitis of both medium and large-sized vessels typically affecting females 50 years of age or older. Severe complications can include permanent visual loss, acute coronary syndrome, or stroke. This case will present an atypical presentation of bilateral OPN which can be a rare manifestation of GCA; **Case Presentation:** Our patient developed acute, painful worsening central vision loss progressing from right eye to left with bilateral extraocular motility restriction and magnetic resonance image (MRI) revealed bilateral, circumferential optic nerve sheath enhancement suggesting optic perineuritis (OPN). Temporal artery biopsy confirmed GCA with bilateral temporal arteritis. The patient was treated with a high dose course of corticosteroids followed by a taper and was started on upadacitinib with symptomatic improvement; **Conclusions:** This case underscores OPN as a rarer manifestation of giant cell arteritis that can present with bilateral painful eye movements and vision loss. Early recognition and prompt corticosteroid therapy are essential to prevent irreversible visual impairment.

## 1. Introduction and Clinical Significance

Giant cell arteritis (GCA) is a rare (incidence of 10 in 100,000) autoimmune vasculitis affecting medium- and large-sized arteries [1,2]. GCA is frequently associated with significant debilitating features such as vision loss and jaw claudication [1,2]. The classical demographic is Northern European females older than 50 years and clinical manifestations can include constitutional symptoms such as fever, weight loss, headache. Depending upon the site of arterial inflammation, symptoms can include temple tenderness, jaw claudication, diplopia, vision loss, or even symptoms of acute coronary syndrome [3]. If left untreated, vision loss can become permanent in twenty to thirty percent (20–30%) of patients [4]. Thus, it is vital to recognize the clinical presentation so that immediate immuno-suppressive therapy can begin prior to diagnostic confirmation with temporal artery biopsy [5].

Atypical presentations of GCA include manifestations such as optic perineuritis (OPN) [6,7,8]. Prior studies on GCA patients have revealed that while between 10% and 23% of patients present with vision changes, only 0.5–1% present with an initial onset of OPN [9,10], leading to a poor definition for this particular disease presentation [11,12]. The typical OPN presentation involves bilateral painful extraocular movements (EOMs) and bilateral vision loss [3,13]. In a case series by Eldaya et al., in patients with a pathology with confirmed giant cell arteritis, seven of those patients (over half) also had optic nerve perineuritis, which may indicate possible under-diagnosis of OPN [1]. However, the literature on global etiologies of OPN, including two case series, only revealed a small portion of OPN cases being secondary to GCA, and more common etiologies of OPN include idiopathic (with almost always unilateral presentation), syphilis, sarcoidosis, and anti-myelin oligodendrocyte glycoprotein (MOG) disease [14,15]. The non-GCA presentations of OPN are often painless [16]. Madkhali et al. discuss the unique case report of OPN in a GCA patient, confirming the bilateral and painful presentations associated with vision loss [17].

Given the rare and heterogenuous origin of OPN, it is important to be able to distinguish the idiopathic type from the more insidious secondary OPN with underlying treatable disease such as GCA. Herein, the authors describe a 57-year-old female who initially presented with bilateral painful extraocular motions and central vision loss who was later found to have biopsy positive giant cell arteritis with associated Magnetic Resonance Imaging positive optic nerve perineuritis.

## 2. Case Presentation

### 2.1. Initial History and Presentation

The patient was a 57-year-old female with a medical history of hypothyroidism who initially presented to the emergency room (ER) with an acute on subacute right eye central vision loss. The right eye vision loss had been fluctuating for two weeks, but had acutely worsened as central vision loss extended to the left eye. Neurology was consulted for potential stroke. She underwent Computed Tomography (CT) head and Computed Tomography Angiogram (CTA) of head and neck which was negative for acute stroke, acute hemorrhage, aneurysm, vascular malformation, or large vessel occlusion.

On further questioning, the patient described several days of tenderness and pressure along the bilateral temples and orbits of the eyes, painful extraocular motions, bilateral jaw tenderness exacerbated by chewing, and dyschromatopsia. She denied vertical or horizontal diplopia. Prior to presentation, she visited an urgent care clinic and a clinician there diagnosed her with sinusitis and subsequently prescribed prednisone and amoxicillin for a presumed infection. She reported that orbital pressure improved with steroids, but that her visual deficits remained.

On the ocular exam, the conjunctiva and corneas were clear, extraocular motions were within normal limits, and the pupils were equal, round, and reactive. Relative afferent pupillary defect was absent bilaterally in the eyes. The funduscopic exam was negative for optic disk edema or atrophy with a cup-to-disk ratio of less than 0.5 and no appreciable vascular abnormalities. The intraocular pressure of the right eye was 16 mmHg, and of the left eye was 14 mmHg (normal 10–21 mmHg). Unfortunately, in our center, ophthalmology does not regularly come to the emergency room or inpatient wards and therefore was not able to come to visit the patient while inpatient.

The differential diagnosis upon initial history and physical included Multiple Sclerosis, Neuromyelitis Optica Spectrum Disorder, Myelin Oligodendrocyte Glycoprotein Antibody-Associated Disease, GCA or other vasculitides, idiopathic perineuritis, idiopathic optic neuritis, central/branched retinal artery occlusion, syphilis, sarcoidosis, or infection of the orbits. The presence of painful EOMs was suggestive of an optic neuritis or perineuritis, but a history of jaw claudication and temporal tenderness on palpation favored GCA or other vasculitidies. Other secondary causes of inflammation were thought to be less likely, given lack of historical or exam findings. While other vasculitides such as Polyarteritis Nodosa (PAN) were considered, due to the classical nature of the patient’s age and demographics, the major concern was that of giant cell arteritis.

### 2.2. Laboratory Findings

Initial labs revealed elevated erythrocyte sedimentation rate (ESR) at 70 mm/h, elevated C-reactive protein (CRP) at 26.8 mg/L, and elevated anti-nuclear antibody titer at 1:160. Lumbar puncture was conducted two days after initial admission with the following abnormally high cerebrospinal fluid labs: 8 white blood cells/uL, 972 red blood cells/uL, spinal fluid (SF) neutrophil percent 33%, and SF glucose 118 mg/dL. However, the lymphocyte percentage was normal at 56%, the monocytes/macrophages percentage was low at 11%, protein was normal at 37.7 mg/dL, and the IgG synthesis rate in the SF was −4.8 mg/24 h which is well within the normal range. Oligoclonal bands were absent, myelin basic protein was normal at <2.0 mcg/L, SF IgG was normal at 2.1 mg/dL, and SF and serum MOG antibody was negative. SF and serum aquaporin 4 IgG was negative. The spinal fluid was cultured and demonstrated no growth on the media. Thyroid stimulating hormone was normal at 2.32 mIU/mL, anti-thyroid peroxidase antibodies in the serum were 13.7 IU/mL and Thyroglobulin antibodies in the serum were 2.2 IU/mL, both within normal limits. MOG antibodies and AQP-4 antibodies in the serum were negative. IgG4 was normal at 24.6 mg/dL, and the screening for perinuclear antineutrophil cytoplasmic antibodies (P-ANCA) and cytoplasmic antineutrophil cytoplasmic antibodies (C-ANCA) was negative (Table 1).

The initial complete serum blood count was unremarkable. Urinalysis was unremarkable for infection and procalcitonin was normal at 0.03 ng/mL (Table 1). Screening for tuberculosis, syphilis, Coccidioides, varicella virus, herpes simplex virus, hepatitis B, and hepatitis C was negative (Table 1).

### 2.3. Radiographic and Diagnostic Findings

MRI of the brain and orbits with and without contrast demonstrated T2 hyperintensities of bilateral optic nerve sheaths extending into post-septal intraconal adipose tissue (Figure 1). The ultrasound arterial duplex of the left and right superior temporal arteries and axillary arteries was negative for abnormal velocities. Bilateral temporal artery biopsies were performed by vascular surgery and histopathological findings demonstrated luminal narrowing, thickened intimal lamina, disruption of internal elastic lamina, and lympho-plasma histiocytic aggregates consistent with a final diagnosis of giant cell arteritis (Figure 2 and Figure 3).

### 2.4. Treatment and Patient Progression

Given the concerning clinical presentation of GCA, methylprednisolone 1000 mg was immediately initiated which the patient received for a total duration of four days while inpatient. On hospital discharge, the patient was started on a prednisone taper for six weeks. On outpatient follow-up, nine days later, the patient reported significant improvement in visual symptoms, which had nearly resolved except for some minor intermittent blurry vision. Pain with ocular movements resolved, but jaw claudication was still present, although improved from prior severity before steroid treatment. GCA was chosen as the diagnosis over other medium vessel vasculitides due to the pathology results, the age of the patient, the lack of other systemic symptoms such as weight loss or fever, the presence of classical symptoms of GCA such as jaw claudication, and lastly, the lack of other infectious causes which might precipitate other vasculitides such as hepatitis B or C, tuberculosis, syphilis, or coccidioides. During the steroid taper, she was started on upadacitinib 15 mg daily. This medication was chosen as it is a once-a-day, oral medication that is FDA-approved for the treatment of giant cell arteritis and is considered to be one of the standard of care medications for this disorder.

During her follow-up visit with Neuro-ophthalmology, the patient underwent a number of ophthalmologic tests including visual acuity testing which demonstrated normal visual acuity at 20/20 vision bilaterally. Ishihara plates were utilized for color vision with the patient getting 13/14 correct in the right eye and 11/14 correct in the left eye. Slit lamp testing and funduscopic testing were well within normal limits without evidence of structural damage or abnormalities in the corneal, lens, or the structures of the retina. Lastly, Optical Coherence Tomography of the retinal nerve fiber layer (RNFL) and macular ganglion cell layer (GCL) did not demonstrate any focal thinning bilaterally.

Since discharge, the patient has remained symptom-free on the upadacitinib 15 mg daily. She is approaching, at the time of writing, nearly one year since her initial flare-up of giant cell arteritis. She was deeply appreciative of the care of the Neurology, Rheumatology, Vascular surgery, and Pathology services. She has followed up with Neurology, Neuro-ophthalmology, and Rheumatology and sees Rheumatology with fairly regularly at around 3-month interval appointments at present. A table of the major clinical event can be found in the Supplimentary Materials (Table S1).

## 3. Discussion

GCA is the most common systemic vasculitis in adults over the age of 50 and carries a high risk of irreversible vision loss if not treated early. Classic presentations include new temporal headache, scalp tenderness, jaw claudication, polymyalgia rheumatica, and anterior ischemic optic neuropathy. About twenty to twenty-five percent (20–25%) of patients present with atypical or occult symptoms that can delay diagnosis. One important ophthalmic manifestation is optic peri-neuritis (OPN) which is an inflammatory condition involving the optic nerve sheath rather than the axon [17].

OPN is often idiopathic but can also be linked to systemic infectious or autoimmune conditions such as an IgG4-related disease, tuberculosis, syphilis, sarcoidosis, or lymphoma [7,18]. Unlike demyelinating optic neuritis which causes central vision loss and is associated with Multiple Sclerosis, OPN typically presents with peripheral visual field deficits and orbital pain worsened by extraocular motions. The key MRI feature is circumferential enhancement of the optic nerve sheath with sparing of the nerve itself described as the “tram track” or “doughnut” sign. OPN usually responds to corticosteroids, but relapses are common especially if secondary to underlying systemic disease [19,20,21]. OPN has been described secondary to Polyarteritis Nodosa [22], Graves’ disease, granulomatosis with polyangiitis (GPA), systemic lupus erythematosus (SLE), Immunoglobulin G4-related disease (IgG-4 RD), Rheumatoid arthritis, Sarcoidosis, Behcet’s disease, scleroderma, and gout [23]. In cases of Polyarteritis Nodosa there are reports of retinal vasculitis and central retinal artery occlusion [24], diplopia [25], and ocular motility disturbances [26] which can mimic classical symptoms of optic perineuritis but these clinical findings are distinctly different than OPN.

In a literature review, only a few cases describe OPN as a manifestation of biopsy-proven GCA. In the case report, by Madkhali et al. [17], OPN characterized by non-specific vision abnormalities in both eyes and pain with extra ocular motion pain proceeded anterior ischemic neuropathy and in the case series by Eldaya et al. they describe the vision loss as a loss of visual acuity unilaterally in all of the cases of biopsy positive GCA [1]. However, in our case the patient had acute painful, central vision loss on bedside visual field test that fluctuated and action was taken quickly enough to prevent permanent optic nerve damage or visual acuity loss when she was tested by neuro-ophthalmology in the outpatient. The bilateral involvement and associated cranial nerve dysfunction are unusual and expand on prior reports that largely described unilateral cases [1]. The combination of OPN and biopsy-proven GCA supports the hypothesis that perivascular inflammation in GCA can extend beyond large and medium arteries to involve orbital structures.

## 4. Conclusions

Clinicians should consider GCA in older adults presenting with MRI evidence of OPN when orbital pain and motility restriction are present. Early corticosteroid initiation is crucial to prevent progression to anterior ischemic optic neuropathy and irreversible vision loss. Temporal artery biopsy and vascular imaging remain essential to confirm the diagnosis and rule out other inflammatory or infectious causes of OPN.

## Figures and Tables

**Figure 1:**
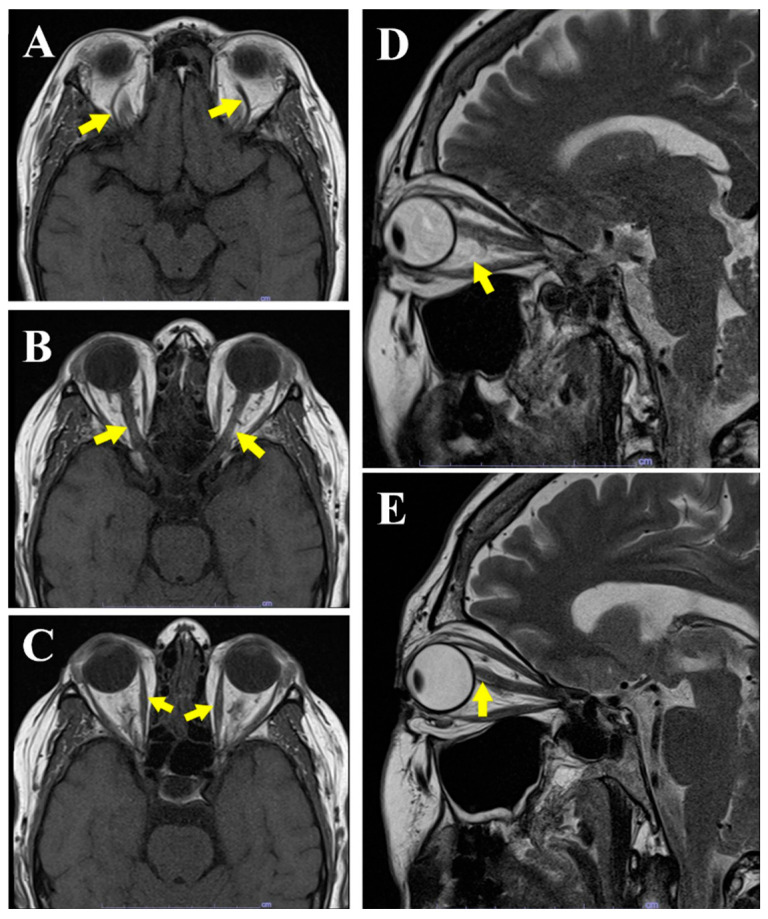
Magnetic Resonance Imaging of the brain and orbits. (**A**) is an axial T1 weighted image that demonstrates normal vasculature. (**B**) is an axial T1 weighted image that demonstrates normal optic nerves. (**C**) is an axial T1 weighted image that demonstrates normal extraocular musculature and peri-optic nerve hyperintensity with signs of inflammation. (**D**) is a Sagittal T2 weighted imaging of right orbit and (**E**) is a Sagittal T2 weighted imaging of left orbit showing the extent of optic sheath hyperintensity and peri-neuritis compared to the optic nerve itself.

**Figure 2:**
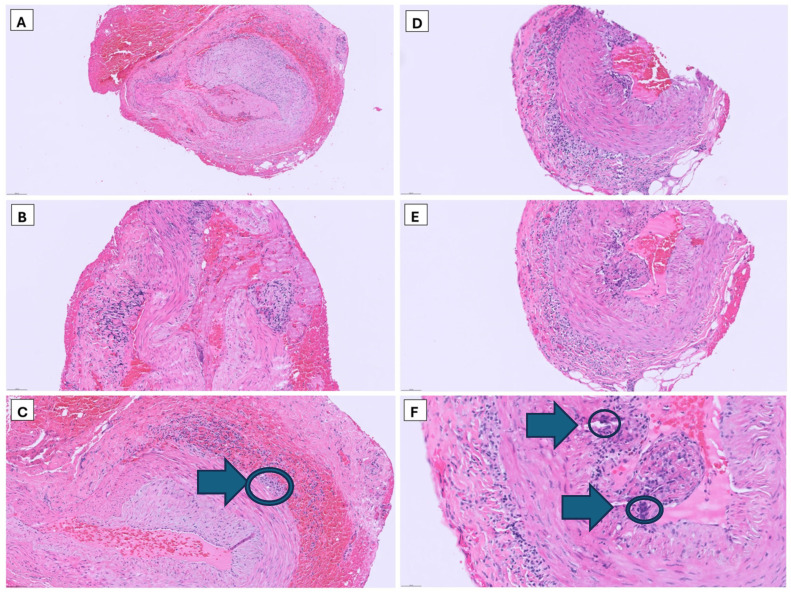
First sample revealing focal inflammation with lymphocytes and histiocytes infiltrating the wall of the artery 10× zoom (**A**,**B**) and 20× zoom (**C**), demarcated on (**C**) with a blue arrow and circle; second sample also revealing the histiocytes infiltrating in the internal layer of the vessel 10× zoom (**D**,**E**) and 20× zoom (**F**), demarcated on (**F**) with blue circles and blue arrows highlighting the classical “Giant Cells” present on pathology.

**Figure 3:**
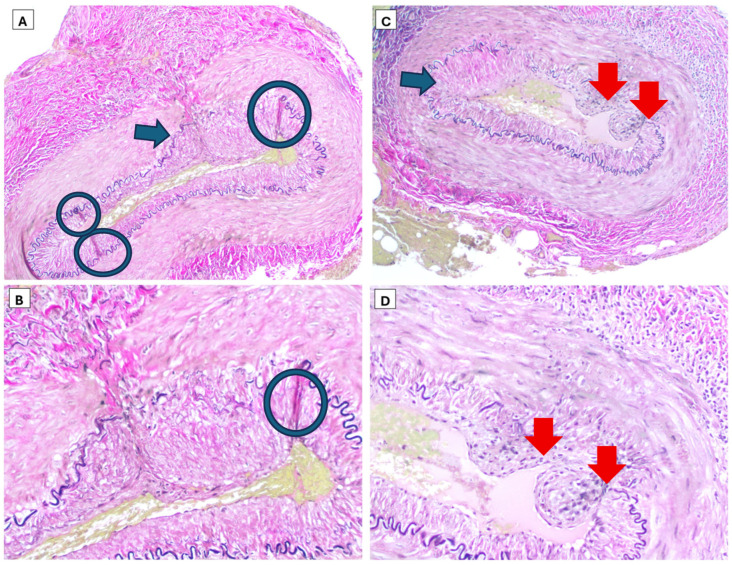
Elastic stain of the first sample 10× zoom (**A**) and 20× zoom (**B**); elastic stain of the second sample 10× zoom (**C**) and 20× zoom (**D**). Circles mark the disruption of the internal elastic lamina. Thick red arrows demonstrate luminal narrowing. Thick blue arrows indicate intimal thickening.

**Table 1:** Patient’s laboratory results.

Lab	Result	Reference Range
**Hematology**		
WBC	9.8 K/uL	4–11 K/uL
Neutrophils	5.81 K/uL	1.50–7.80 K/uL
Hemoglobin	13.9 g/dL	12.0–16.0 g/dL
Platelets	349 K/uL	130–450 K/uL
ESR	70 mm/h	<30 mm/h
CRP	26.8 mg/L	<4.9 mg/L
Procalcitonin	0.03 ng/mL	<0.24 ng/mL
**Infectious Workup**		
Sputum Culture	Negative	Negative
Spine Fluid Culture	Negative	Negative
T-Spot TB	Negative	Negative
HBV Core Ab	Negative	Negative
HCV Ab Screen	Negative	Negative
VDRL CSF	Negative	Negative
Syphilis antibody	Negative	Negative
Coccidioides antibody IgM	Negative	Negative
Coccidioides antibody IgG	Negative	Negative
VZV PCR	Negative	Negative
HSV 1 PCR	Negative	Negative
HSV 2 PCR	Negative	Negative
**Serology**		
ANA Titer	1:160	<1:40
C-ANCA	Negative	Negative
P-ANCA	Negative	Negative
IgG	1180 mg/dL	600–1640 mg/dL
IgG4	24.6 mg/dL	4–86 mg/dL
MOG antibody	Negative	Negative
AQP4 NMO IgG	Negative	Negative

Acronyms—WBC: white blood cells, ESR: erythrocyte sedimentation rate, CRP: C-reactive protein, T-spot TB: mycobacterium tuberculosis T-spot, HBV core ab: hepatitis B virus core antibody, HCV ab screen: hepatitis C virus antibody screen, VDRL CSF: Venereal Disease Research Laboratory Test cerebrospinal fluid, IgM: immunoglobulin M, IgG: immunoglobulin G, VZV PCR: Varicella zoster virus polymerase chain reaction, HSV 1 PCR: herpes simplex virus polymerase chain reaction, HSV 2 PCR: herpes simplex virus polymerase chain reaction, ANA: antinuclear antibody, C-ANCA: cytoplasmic antineutrophil cytoplasmic antibodies, P-ANCA: perinuclear antineutrophil cytoplasmic antibodies, IgG4: immunoglobulin G4, MOG: myelin oligodendrocyte glycoprotein, and AQP4 NMO IgG: aquaporin 4 neuromyelitis optica immunoglobulin G.

## Data Availability

The original contributions presented in this study are included in the article. Further inquiries can be directed to the corresponding author.

## Supplementary Materials

The following supporting information can be downloaded at: https://www.mdpi.com/article/10.3390/reports9020187/s1, Table S1: Timeline of Events.
