# Supplementary material for: A Rare Case Reveals Important Consideration of the Diagnosis of Giant Cell Arteritis in Patients with Bilateral Painful Optic Perineuritis
Source: Reports (MDPI). 2026 Jun 15;9(2):187. doi: 10.3390/reports9020187 (PMC13307026; doi:10.3390/reports9020187)
Supplement: Supplementary file 1 [file reports-09-00187-s001.zip › reports-4321173-supplementary/Table S1.pdf]

Table S1. Timeline of Events.

Date	2 weeks Prior to Hospitalization (-)14 days	Day of Hospitalization Day 0	The four subsequent days during hospitalization Days 1-4	Post Discharge Days 5-Week 6
Clinical Events	Right eye central vision loss started. Tenderness and pressure along the bilateral temples and orbits of the eyes, painful EOMs, bilateral jaw tenderness exacerbated by chewing, and dyschromatopsia noted by the patient. She visited urgent care and a clinician diagnosed her with sinusitis and was subsequently prescribed prednisone and amoxicillin for presumed infection	Central Vision loss spread to the left eye. Patient underwent CT of the Head and CTA of the head and neck to evaluate for potential stroke. She also underwent laboratory testing for other causes of symptoms.	Underwent MRIs of the Brain and Orbits. Underwent ultrasound arterial duplex of left and right superior temporal arteries and axillary arteries. Lastly was started on 1000 mg of IV Methylprednisolone.	Patient reported improvement of symptoms with starting the Steroid taper when she was seen 9 days after discharge. She continued this steroid taper for 6 total weeks. Following her appointment on the 9th days post discharge she was started on upadacitinib 15 mg daily
